# Host-Pathogen Interactions of Retroviruses

**DOI:** 10.1155/2012/648512

**Published:** 2012-10-24

**Authors:** Abdul A. Waheed, Abraham L. Brass, Suryaram Gummuluru, Gilda Tachedjian

**Affiliations:** ^1^Virus-Cell Interaction Section, HIV Drug Resistance Program, National Cancer Institute at Frederick, Frederick, MD 21702, USA; ^2^Microbiology and Physiological Systems, University of Massachusetts Medical School, Worcester, MA 01655, USA; ^3^Department of Microbiology, Boston University School of Medicine, Boston, MA 02118, USA; ^4^Center for Virology, Burnet Institute, Melbourne, VIC 3004, Australia; ^5^Department of Microbiology, Monash University, Clayton, VIC 3168, Australia; ^6^Department of Medicine, Monash University, Melbourne, VIC 3004, Australia

Retroviruses, such as HIV-1, are enveloped RNA viruses that use the enzyme reverse transcriptase (RT) to make a DNA copy of their RNA genome during replication in the host cell. The retrovirus life cycle is generally divided into two distinct phases: the early and late phase. The early phase encompasses virion entry into the host cell, reverse transcription of the viral RNA, nuclear import of the pre-integration complex (PIC), and integration of viral DNA into the host chromosome. The late phase involves transcription of viral DNA to multiple copies of viral RNA, translation of viral proteins, trafficking of viral proteins and genome to assembly sites, budding of viral particles, and, finally, maturation. A number of host factors have been implicated in specific steps of virus replication, and identification of such factors is a rapidly growing field. Recently, many host proteins were identified in genome-wide siRNA screens as being required for HIV-1 replication [1–3].

Over 25 antiretroviral drugs are currently in clinical use for treating HIV-1, and except for the fusion inhibitor that targets the viral envelope glycoprotein gp41 or the coreceptor CCR5, these drugs target the activity of the viral enzymes RT, integrase (IN), and protease (PR) [4–8]. The advent of highly active antiretroviral therapy (HAART) has made a significant impact on the natural history of HIV/AIDS by dramatically prolonging the life of HIV-infected individuals [9]. However, besides long-term drug toxicity and drug-drug interactions leading to treatment failures, significant limitations of antiviral therapy include the emergence of drug-resistant viral variants [10]. Further, the success of topical and oral preexposure prophylaxis (PrEP) in preventing the sexual transmission of HIV in a clinical trial setting presents potential concern because antiretrovirals or drugs with similar resistance profiles are used both for therapy and prevention [11]. This, in a PrEP setting, could either result in the transmission of drug-resistant viral strains or the generation of such viral strains in individuals taking PrEP unaware of their HIV infection status, thereby limiting future therapeutic options. Such concerns warrant efforts to identify novel inhibitors of HIV. Understanding the role of host proteins in viral replication could potentially lead to the development of new therapeutic strategies to combat this deadly pathogen.

This special issue brings together 17 reviews by experts on various aspects of the HIV-1 life cycle, highlighting the significant roles played by host factors in virus replication, and the antiviral agents that act on the viral and cellular targets. These reviews do not necessarily represent an exhaustive inventory of the current state of research or opinion in the field. Instead, the reviews cover the widely studied host-factors in each step of the HIV-1 replication cycle and antiviral therapy targeting viable cellular and viral targets. We, the guest editors, would like to sincerely thank all the authors for their contribution to this special issue and the reviewers for their time and expertise.

In his review *“TRIM5 and the regulation of HIV-1 infectivity*,*”* Jeremy Luban offers an in-depth analysis of how TRIM5 impedes retroviral infection, including the recent exciting data concerning TRIM5's innate immune signaling capacity that permits the host factor to recognize HIV-1's capsid (CA) lattice and subsequently signal to downstream antiviral effectors. This review also presents a comprehensive picture of a major challenge facing the field today—understanding the structural basis of TRIM5's recognition of HIV-1 CA.

Esposito and colleagues review the structure and function of the HIV-1 RT and the mode of action of nucleoside/nucleotide reverse transcriptase inhibitors (NRTIs) and nonnucleoside reverse transcriptase inhibitors (NNRTIs). The authors discuss novel RT inhibitors that are currently in development, including NRTIs that act as chain terminators and those that act by blocking RT translocation or delaying DNA chain termination. New NNRTIs designed to inhibit HIV-1 mutants resistant to first-generation NNRTIs such as nevirapine and efavirenz, and those that block RT by competing with nucleotide substrate, a mechanism distinct from classical NNRTIs, are also covered in this review. Further, the authors highlight RNaseH inhibitors and pyrophosphate analogues and molecules that disrupt the essential RT subunit interaction.

Sheehy and Erthal in their exceptionally well-written review *“APOBEC3 versus retroviruses, immunity versus invasion: Clash of the Titans”* deftly touch on the major advances in understanding the role of this fascinating antiretroviral protein, and highlight some compelling future topics for research. The authors also cover the latest *in vivo* observations on APOBEC3 functions in HIV-infected patients.

Macrophages are a key source of HIV persistence *in vivo*. The review by Gavegnano and colleagues describes how nucleotide pools differ in macrophages compared to actively dividing T lymphocytes. Specifically, dNTP levels are limited relative to high levels of rNTPs and this disparity, shaped by the myeloid-cell-specific restriction factor SAMHD1, leads to preferential incorporation of rNTPs compared to dNTPs during reverse transcription. The authors discuss how the incorporation of rNTPs in the nascent viral DNA strand, which dispels the dogma that RT can only incorporate dNTPs, can be exploited in the design ribonucleoside chain terminator inhibitors that block HIV replication specifically in macrophages.

Felipe Diaz-Griffero shifts our attention to a host dependency factor that is required for HIV-1 infection by expertly discussing recent advances in elucidating the role of the karyopherin, TNPO3, in lentiviral replication. Specifically, this timely review covers recent genetic and biochemical data showing that the HIV-1's CA protein is the viral determinant for the requirement of TNPO3 during infection. Although the precise role of TNPO3 in lentiviral infection is a hotly debated topic in the field, this review succinctly frames the current state of this discussion, thereby providing a much-needed overview of this fast-moving topic.

The Debyser group has presented a comprehensive overview on HIV-1 dependency factors (HDFs) involved in viral integration and nuclear import. The work primarily discusses LEDGF, an HDF which is critical for mediating lentiviral integration; we are taken from the early days when biochemical approaches implicated LEDGF via its physical association with HIV-1, into the current era, where high-throughput small-molecule screens have identified novel inhibitors of this now-well-established host-viral interaction. The growing field of HIV-1 nuclear import is also covered, demonstrating the exciting work done in this area after a transformative whole genome siRNA screen first catalyzed interest in this fascinating topic. A recapping of the subsequent contributions by many laboratories to determine the mechanism of numerous HDFs in the nuclear import of the virus is also provided.

Schiralli Lester and Henderson in their review masterfully integrate the vast amount of data investigating HIV-1 proviral transcription. With deft skill, the pair interprets the role played by viral transcription in the rapidly expanding field of HIV-1 latency wherein viral reservoirs resist eradication after long-term antiviral therapy. Important topics in proviral transcriptional regulation covered in this selection include host transcriptions factors, chromatin, transcriptional interference, and elongation, the latter with special emphasis on the actions of the viral accessory protein, Tat. Tat is defined as a critical regulator and therapeutic target for the alleviation of latency and a potential cure. Multiple groups in both academia and industry are now reporting their fascinating investigations of HIV-1 latency, thus providing a dynamic stage for this prescient effort.

HIV-1 RNA interacts with numerous proteins including the viral nucleocapsid (NC) protein, and the structure of the RNA genome is linked to HIV-1 replication. However, the higher-order structure of the viral RNA is poorly understood. Sztuba-Solinska and Le Grice have come up with an excellent review on the utilities of the selective 2′-hydroxyl acylation analyzed by primer extension (SHAPE) technology, which can resolve the structure and quantify the flexibility of RNA at single-nucleotide resolution. The authors provide an overview of the SHAPE methodology- and also discuss the benefits and limitations of this technology in studying the structure of short RNAs. Shape technology has enabled the resolution of the structure of the entire HIV-1 genome (~9750 nucleotides) at the single-nucleotide level, and such detailed structural understanding could elevate the viral RNA as a viable target for small-molecule therapeutic intervention.

Expression of the HIV-1 Gag precursor protein, Pr55^Gag^, is sufficient to produce virus-like particles. Nascent Gag traffics to the assembly sites—predominantly the plasma membrane, where Gag multimerization promotes virus budding, and finally the host-mediated scission leads to release of immature particles. Ono and colleagues present a comprehensive review on the dynamic association of Gag with membrane microdomains- and survey the role of lipid rafts and tetraspanin-enriched membrane microdomains in HIV-1 assembly. Besides discussing the more recent understanding of Gag multimer-driven reorganization of membrane microdomains, the authors also highlight the role of plasma membrane microdomains in cell-cell spread through virological synapses in T cells.

Hattleman et al. in their review on retroviral restriction factors, *“TRIM22: a diverse and dynamic antiviral protein,”* investigate another fascinating TRIM family member, TRIM22. The authors first relate TRIM22's evolutionary history including gene expansion/loss and the evidence revealing that the gene has experienced strong positive selection. Interestingly, the authors describe the growing list of viruses restricted by TRIM22, including encephalomyocarditis virus, hepatitis B virus, and HIV-1. Lastly, the authors focus on the latest developments in the cell biology of TRIM22, including its role in cell proliferation and differentiation, and in cancer and autoimmune disease.

HIV-1 Gag, via the C-terminal PTAP motif known as the “late domain” hijacks the cellular protein Tsg101, a component of endosomal sorting complexes required for transport (ESCRT-1) complex during virus budding. Erlich and Carter review the role of ESCRT and non-ESCRT proteins in virus budding and release. The authors describe the role of PI(4,5)P2 in Gag targeting to the plasma membrane and the late domain-mediated recruitment of ESCRT machinery in HIV-1 budding. Recently, the Carter Group reported the activation of the inositol 1,4,5-triphosphate receptor (IP3R), which gates intracellular calcium ion stores, as a determinant in Gag trafficking and virus release.

Hammonds, Wang and Spearman provide an excellent state-of-the-art overview of the rapidly advancing field of tetherin biology, with a focus on recent advances in the understanding of the structure and function of this transmembrane protein. The authors begin by describing the historical details of the relationship between tetherin and the HIV-1 accessory factor, Vpu, and then discuss the relevance of tetherin in the replication and spread of other retroviruses. Further, the authors present a balanced synopsis of evidence for and against the model that proposes tetherin localization to membrane microdomains as a critical determinant of its antiretroviral activity.

The Env glycoprotein associates with Gag during virus assembly to form infectious virus particles. Murakami in his review describes the biosyntheseis, trafficking, and incorporation of Env glycoproteins into virus particles. In this review, he surveys various proposed models for Env incorporation into virus particles. The Env incorporation can be passive or via direct or indirect Gag-Env interaction, which reportedly occurs at specific membrane microdomains and is mediated by specific host factors. Murakami's review covers in detail the host cellular factors implicated in Gag-Env interactions and their specific role in virion incorporation.

The HIV-1 PR activity converts immature particles to infectious mature particles. In her review, Adamson details the sequential cascade of events that accompany the PR- mediated cleavage of the Gag polyprotein. Inhibiting PR activity by protease inhibitors (PIs) results in the production of noninfectious virus particles, and nine PIs are currently approved for clinical use. In contrast, maturation inhibitors bind to Gag and specifically block the individual cleavage events or alter the order of cleavage events, thereby resulting in the production of aberrant particles. In this review, Adamson provides an overview of the mechanism of action of PIs and maturation inhibitors- and highlights the problems associated with drug-resistant mutants.

In their contribution, Hartman and Buckheit review the HIV inhibitors currently in clinical use, novel HIV RT inhibitors in the pipeline, and drugs that target additional viral proteins including the gp41 involved in viral fusion, the zinc fingers of NC required for viral genome encapsidation and reverse transcription, the IN inhibitors that block insertion of the viral cDNA into the host cell chromosome, and the PIs that target viral maturation. The authors also review molecules that target the HIV-1 regulatory and accessory proteins Tat, Rev, Vpu, Vpr, and Vif. The review also examines strategies for targeting host cells proteins (Tsg101 and LEDGF/p75) that are hijacked by HIV for replication, and ways to exploit intracellular host cell restriction factors (i.e., APOBEC3 and tetherin) that block HIV replication. Immunotherapy, gene therapy, and strategies to eliminate the latent reservoirs of HIV are also described.

Microbicides are chemical entities formulated in a gel, cream, ring, film, or tablet that can prevent or reduce transmission of sexually transmitted infections including HIV infection, when applied to the vagina or rectum. In their review, Buckheit and Buckheit provide a comprehensive assessment of the HIV microbicide field and the preclinical tests that are required for progression of a candidate microbicide through the development pathway. The authors also highlight gaps that exist in product development that relate to product dosing, formulation and delivery, and pharmacokinetics and pharmacodynamics, which all must be addressed to improve prioritization of candidate microbicides for clinical testing. Besides vaginal microbicides, the development and formulation of dual compartment use microbicides for both vaginal and rectal use are discussed. The emerging area of multipurpose prevention technologies with the premise to prevent unplanned pregnancies, HIV, and other sexually transmitted infections that can increase HIV acquisition are also described.

A consequence of suboptimal antiretroviral therapy is the emergence of drug-resistant strains of HIV-1, which can lead to therapy failure. Much of our knowledge regarding the type of mutations that emerge during therapy and their role in decreasing drug susceptibility is derived from studies with HIV-1 subtype B. However, 90% of HIV-infected individuals worldwide harbour nonsubtype B variants that contain distinct polymorphisms. Wainberg and Brenner review the ability of such polymorphisms in nonsubtype B HIV to affect the level of resistance mediated by major drug-resistance mutations, and to modulate the evolution of certain drug resistance mutations in the presence of drug. The authors also propose studies that would increase our understanding of the role of polymorphisms in drug resistance and, thereby, promote more informed use of first, second and third-line antiretroviral drugs in different geographical settings. 

There are few research areas that are not covered explicitly in this special issue, such as retrovirus entry, and the role of receptors and coreceptors in virus entry. However, this issue offers a comprehensive view of our understanding of the HIV-1 life cycle, host factors involved in virus replication, and viral and cellular antiviral drug targets.

*Abdul A. Waheed* *Abdul A. Waheed* *Abraham L. Brass* *Abraham L. Brass* *Suryaram Gummuluru* *Suryaram Gummuluru* *Gilda Tachedjian* *Gilda Tachedjian*
